# RP-18 TLC Chromatographic and Computational Study of Skin Permeability of Steroids

**DOI:** 10.3390/ph14070600

**Published:** 2021-06-22

**Authors:** Anna W. Weronika Sobanska, Jeremy Robertson, Elżbieta Brzezińska

**Affiliations:** 1 Department of Analytical Chemistry, Faculty of Pharmacy, Medical University of Lodz, ul. Muszyńskiego 1, 90-151 Łódź, Poland; elzbieta.brzezinska@umed.lodz.pl; 2 Chemistry Research Laboratory, Department of Chemistry, University of Oxford, Mansfield Road, Oxford OX1 3TA, UK; jeremy.robertson@chem.ox.ac.uk

**Keywords:** steroids, skin permeability, thin layer chromatography, calculated physicochemical descriptors

## Abstract

The skin permeability of steroids, as investigated in this study, is important because some of these compounds are, or could, be used in preparations applied topically. Several models of skin permeability, involving thin layer chromatographic and calculated descriptors, were generated and validated using ***K_p_*** reference values obtained in silico and then tested on a group of solutes whose experimental ***K_p_*** values could be found (log ***K_p_***^exp^). The study established that the most applicable log ***K_p_*** model is based on RP-18 thin layer chromatographic data (***R_M_***) and the calculated descriptors ***V_M_*** (molar volume) and ***PSA*** (polar surface area). Two less efficient, yet simple, equations based on ***PSA*** or ***V_M_*** combined with ***HD*** (H-donor count) can be used with caution for rapid, rough estimations of compounds’ skin permeability prior to their chemical synthesis.

## 1. Introduction

Steroids are an important class of pharmaceutical actives which may be administered by different routes, including transdermal delivery [1]. Their skin permeation has been a subject of interest for a relatively long time [2,3,4]. In addition to experimental studies of steroids’ ability to cross the skin barrier, attempts have been made to predict this property in silico. However, due to their polyfunctionality and relatively large molecular volumes, steroids are significantly different from many substances whose skin permeability has been studied, and not all the known algorithms of skin permeability are suitable for this group of solutes [4]. 

The rate of a molecule’s permeation through skin is expressed as the flux (***J***), which is the amount of substance permeated per unit area and unit time. The flux depends on the permeability of the skin to the permeant (***K_p_***) and the gradient of permeant concentration across the skin (Δ***c***):

***J*** = ***K_p_*** ∙ Δ***c***

For passive diffusion, the permeability coefficient ***K_p_*** depends, in turn, on the partition coefficient ***P***, the diffusion coefficient ***D*** and the diffusional path length ***h***:

$$K_{p}=\frac{P\cdot D}{h}$$

Transdermal permeation of drugs may be studied using many techniques, including in vitro permeation experiments on excised human skin [5], animal skin, cultured human skin cells or synthetic membranes [5,6]. It is also known that skin permeation correlates with some easily obtained physicochemical parameters of a molecule, including log ***P_ow_***, which is the partition coefficient between octanol and water and a well-established predictor of a compound’s lipophilicity and biological activity [7]. However, it has been demonstrated that log ***P_ow_*** is not applicable as a single measure of log ***K_p_*** across a very wide range of chemical families, so molecular weight (***M_w_***) or volume (***V_M_***), hydrogen bond donor and acceptor activity (***H_d_*** and ***H_a_***, respectively), and melting point (***M_Pt_***) values are incorporated as additional descriptors [8,9,10,11,12,13,14]. Different computational skin permeability models have been reviewed and compared by several authors [3,15,16,17,18,19,20].

Liquid chromatography is frequently used to investigate physicochemical properties and biological activity of solutes, including their skin permeability. The chromatographic techniques used to predict the ability of molecules to cross the skin barrier include normal and reversed-phase thin layer chromatography [21,22], immobilized artificial membrane (IAM) column chromatography [23,24,25,26], RP-18 column chromatography [24,25], column chromatography on a unique stationary phase based on immobilized keratin [27], and biopartitioning micellar chromatography (BMC) [28,29,30]. The skin permeability coefficient ***K_p_*** is connected with the chromatographic retention parameters log ***k*** or ***R_M_^0^*** (obtained for column and thin layer chromatography, respectively) via linear or reverse parabolic relationships [22,26]. Chromatographic retention parameters are used either as sole skin permeability predictors, or they are combined with additional descriptors (log ***P_ow_***, ***V_M_***, ***M_w_*** or ***M_Pt_***) [23,24,28,30].

Transdermal drug delivery is an important strategy employed to improve the bioavailability of drugs whose administration by other routes suffers from limitations such as poor drug stability in the gastrointestinal tract, poor permeability through the intestinal membrane or problems caused by first pass metabolism [31]. Although oral delivery remains to date the preferred method of drug administration, transdermal drug delivery systems are gaining in popularity [18,32]. Skin permeability, expressed by the coefficient ***K_p_***, is an important parameter affecting the systemic uptake of drugs after transdermal delivery. The objective of this study was to examine the relationships between the skin permeability coefficient ***K_p_*** and calculated and RP-18 TLC-chromatographic descriptors for a group of steroid drugs acting upon different therapeutic targets. Descriptors derived from the RP-18 thin layer chromatographic system used in this study have appeared in previous works on blood-brain barrier (BBB) permeability [33,34,35] and skin permeation [36] and, according to [37], in some instances the RP-18 TLC retention parameters are better predictors of biological activity than the RP-18 HPLC data. 

## 2. Results and Discussion

The skin permeability coefficient (***K_p_***) is an important parameter that helps in the assessment of a compound’s epidermal permeability; however, the experimentally determined values of ***K_p_*** are available for only some of the drugs within the studied group. For this reason, it was decided that models of skin permeability based on thin layer chromatographic and calculated descriptors should be generated and validated using ***K_p_*** values obtained in silico, then tested on a group of solutes whose experimental ***K_p_*** values could be found (log ***K_p_***^exp^). The estimation methodology used in this study is based on the approaches A to C (Table 1).

A. Equation (1), developed and validated in our earlier research [36]:
  log ***K_p_***^(1)^ = −1.39 (±0.18) − 0.35 (±0.03) (***N*** + ***O***) + 0.15 (±0.04) log ***D*** − 0.23 (±0.06) ***HD***
  (*n* = 60, R^2^ = 0.83, R^2^_adj_. = 0.82, F = 92.3, *p* < 0.01, s_e_ = 0.44) (1)
B. EpiSuite software (DERMWIN v. 2 module) (log ***K_p_***^EPI^), recommended by the US Environmental Protection Agency and related to the widely recognized Potts’ model of skin permeability [10]:
  log ***K_p_*** = −2.80 + 0.66 log ***P_ow_*** − 0.0056 ***M_w_*** (R^2^ = 0.66) (2)
C. PreADMET 2.0 software [38] (log ***K_p_***^pre^)

Initially, attention was turned to partition phenomena in the human stratum corneum. It was noted that Equation (1) may be a source of valuable information on solute partitioning between water and the stratum corneum. The process of skin absorption of topically applied compounds is relatively complex and consists of three steps: (i) penetration of the stratum corneum (SC), either by polar or lipid transport pathways; (ii) permeation through deeper skin layers and (iii) resorption, i.e., the uptake of a substance into the vascular system [39]. The SC is the rate-limiting skin layer [39,40] and good partition between water and the SC is an important prerequisite for effective skin absorption. Skin permeability coefficients calculated according to Equation (1) were correlated with experimental values of SC/water partition coefficients for lipid and protein domains (log ***K_sc/w_***^lip^ and log ***K_sc_***_/w_^prot^, respectively) determined by Anderson et al. [40]. The correlations obtained for a group of hydrocortisone esters (compounds **17** to **27**) were moderate (R^2^ = 0.70 for lipid and 0.41 for protein domain, respectively). A group of 14 other steroid compounds (**2**, **3**, **5** to **16**), whose SC/water and lipid/water partition parameters were studied by other authors [2,41], showed good correlations between log ***K_sc/w_*** and log ***K_p_***^(1)^ (R^2^ = 0.80, *n* = 14). For compounds **3**, **6**, **7**, **12** and **14**, the correlation between log ***K_sc/w_***^lip^ and log ***K_p_***^(1)^ was also linear (R^2^ = 0.85, *n* = 5). 

**Figure 1:**
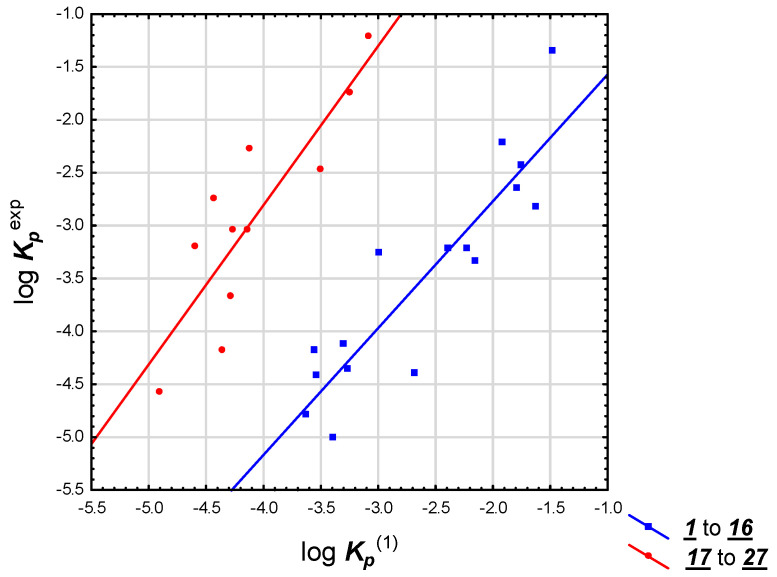
log ***K_p_*** experimental values vs. Equation (1), compounds **1** to **27**.

Equation (1) was applied to a group of 27 steroid drugs whose experimental skin permeability coefficients are available (Table 1). It was discovered that these drugs formed two subgroups (Figure 1): compounds **1** to **16** (log ***K_p_***^exp^ taken from Refs. [2,4,42,43,44]) and **17** to **27** (log ***K_p_***^exp^ given by Anderson et al. [40]). The skin permeability coefficients calculated for these compounds according to Equation (1) (log ***K_p_***^(1)^) were in good agreement with the experimental values (log ***K_p_***^exp^) (linear relationships within the subgroups, R^2^ = 0.81 for compounds **1** to **16** and 0.74 for compounds **17** to **27**, respectively). The correlation between calculated (Equation (1)) and experimental values of log ***K_p_*** for compounds **17** to **27** was even better (R^2^ = 0.84) once two ionic molecules that contain free carboxyl groups (**20** and **21**) were removed as outliers.

**Figure 2:**
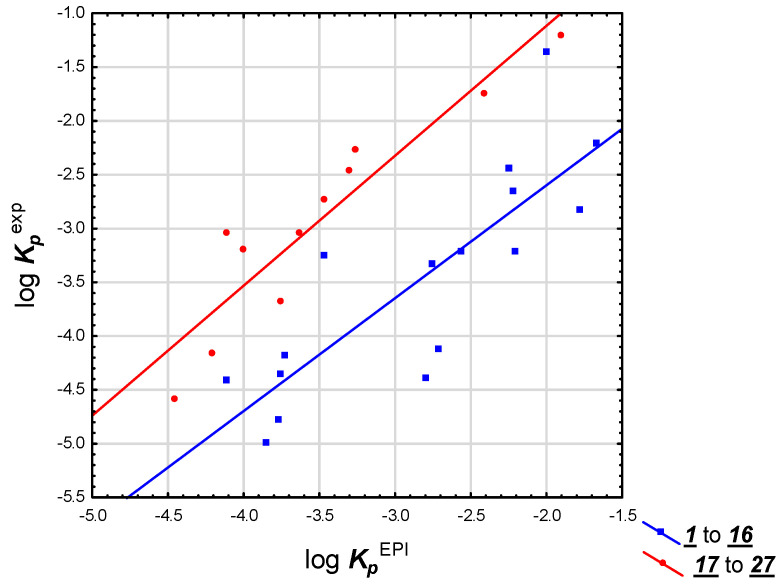
log ***K_p_*** experimental values vs. EpiSuite, compounds **1** to **27**.

A similar situation arose when log ***K_p_***^EPI^ values were considered; thus, compounds **1** to **27** again formed two subgroups (**1** to **16** and **17** to **27**) whose experimental log ***K_p_*** values gave reasonable correlations with log ***K_p_***^EPI^ (R^2^ = 0.69 and 0.86, respectively), although the subgroups partially overlapped (Figure 2). The reasons for discrepancies between experimental log ***K_p_***^exp^ values for compounds **1** to **16** and **17** to **27** are unclear. However, because the log ***K_p_***^exp^ values for compounds **17** to **27** were taken from a single source [40], the differences in experimental methodology may have had more influence on log ***K_p_***^exp^ values obtained by different authors than the physicochemical properties of the studied compounds. Related problems with the “Anderson’s dataset” (with a similar explanation) were described by Abraham et al. [4].

**Figure 3:**
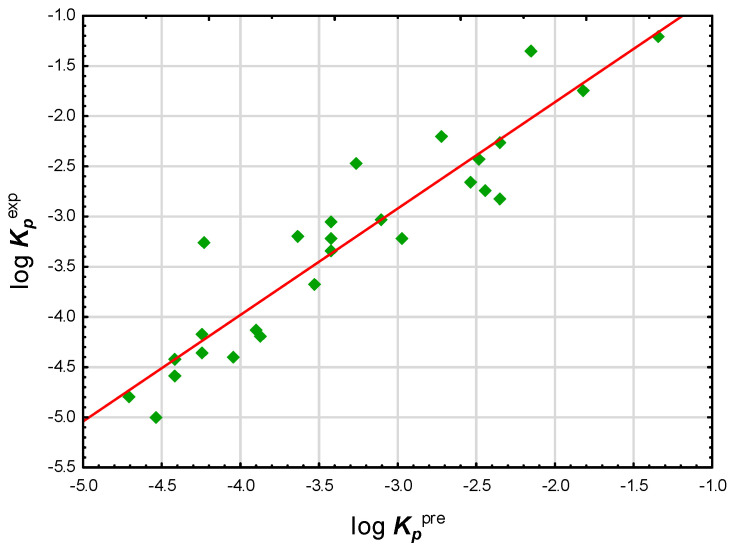
log ***K_p_*** experimental values vs. preADMET, compounds **1** to **27**.

The results of log ***K_p_*** calculations using preADMET software seemed more consistent (Figure 3); compounds **1** to **27** gave a single group whose calculated (log ***K_p_***^pre^) and experimental (log ***K_p_***^exp^) values were in good agreement (R^2^ = 0.87, *n* = 27). However, since there was no reason to suspect that, for studied compounds, the predicted values of log ***K_p_***^pre^ were more (or less) reliable than the values calculated by other methods, the decision was made to consider also log ***K_p_***^EPI^ and log ***K_p_***^(1)^ as reference values in further investigations. 

**Figure 4:**
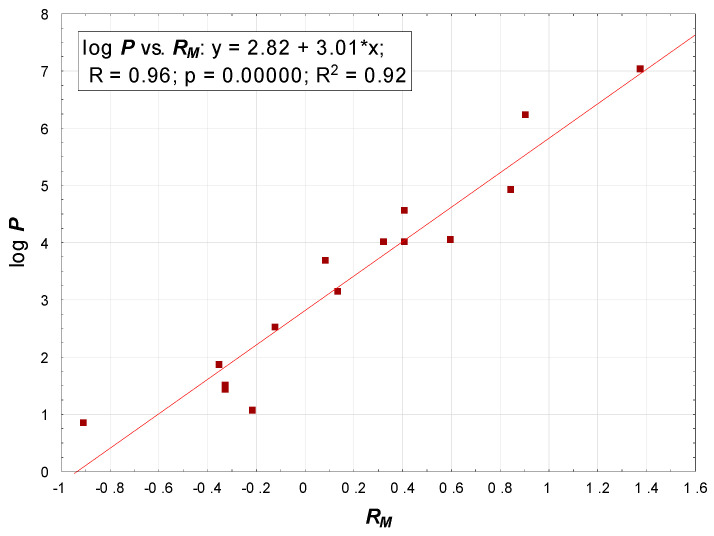
Correlation between calculated log ***P*** and ***R_M_***.

One of the key properties responsible for skin permeability of solutes is lipophilicity. Some earlier chromatographic studies of lipophilicity of steroids and steroid analogues [45,46] were based on the linear extrapolation approach. Chromatographic parameters for a single-solvent mobile phase were obtained by using a series of chromatographic experiments with mobile phases containing different concentrations ***φ*** of a modifier. Plots of ***R_M_*** or log ***k*** (for TLC and HPLC, respectively) vs. ***φ*** were extrapolated to zero concentration of the modifier to furnish ***R_M_*^0^** (log ***k*_0_**). The most common method to do so is by using the linear Soczewiński-Wachmeister equation: ***R_M_*** = ***R_M_*^0^** + ***Sφ*** [47]. Apart from the ***R_M_*^0^** value, other useful chromatographic descriptors derived from the linear extrapolation method are the slope ***S*** and ***C_0_*** = −***R_M_*^0^**/***S***. The extrapolation method, although commonly used and recognized, has certain drawbacks. Several chromatographic experiments are required and the extrapolated ***R_M_*^0^** values depend on a modifier and its concentration range used to generate ***R_M_*** = *f*(***φ***) plots. In this study, therefore, the single chromatographic run approach was used. It was established that for the 16 steroids analyzed chromatographically, ***R_M_*** values collected using a single concentration of an organic modifier in a mobile phase were very closely related to their lipophilicity. For example, for lipophilicity calculated using ACDLabs v. 8.0 software, the relationship between log ***P*** and ***R_M_*** was linear (R^2^ = 0.92, Figure 4). 

**Figure 5:**
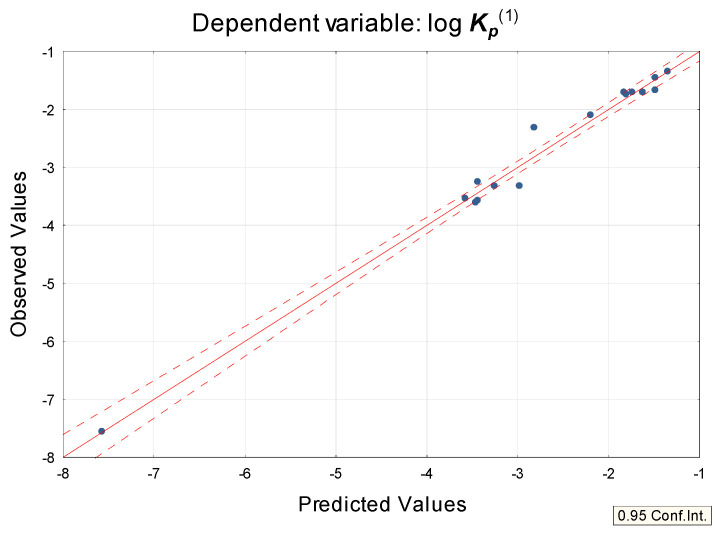
Equation (4) predicted vs. observed values.

Based on log ***K_p_*** reference values obtained by methods A to C, Equations (3)–(5) were developed for compounds **1** to **5** and **28** to **38**, whose RP-18 thin layer chromatographic retention data are available: (Figure 5)

log ***K_p_***^EPI^ = −1.66 (±0.24) − 0.011 (±0.005) ***PSA*** + 0.24 (±0.05) ***HD*** − 0.0036 (±0.0017) ***V_M_*** + 2.01 (±0.24) ***R_M_*** (*n* = 16, R^2^ = 0.99, R^2^_adj_ = 0.98, RMSECV = 0.21, F = 229.0, *p* < 0.01, s_e_ = 0.18) (3)

log ***K_p_***^(1)^ = 0.17 (±0.31) − 0.011 (±0.006) ***PSA*** − 0.14 (±0.06) ***HD*** − 0.0065 (±0.0022) ***V_M_*** + 1.01 (±0.30) ***R_M_*** (*n* = 16, R^2^ = 0.99, R^2^_adj_. = 0.78, RMSECV = 0.31, F = 174.8, *p* < 0.01, s_e_ = 0.22) (4)

log ***K_p_***^pre^ = −3.77 (±0.61) − 0.043 (±0.012) ***PSA*** + 0.18 (±0.13) ***HD*** + 0.011 (±0.004) ***V_M_*** + 0.027 (±0.600) ***R_M_*** (*n* = 16, R^2^ = 0.90, R^2^_adj_.= 0.86, RMSECV = 0.60, F = 23.6, *p* < 0.01, s_e_ = 0.45) (5)

The selection of independent variables in Equations (3)–(5) is a logical consequence of the influence on skin permeability of molecules of lipophilicity, polarity, molecular size and ability to form hydrogen bonds. For example, in Equation (3) the variables were selected by stepwise regression in the following order: ***R_M_*** (which accounts for 89% of total variability), ***V_M_***, ***HD*** and ***PSA***. Equations (3) to (5) were also tested on a subgroup of five compounds analyzed in this study whose chromatographic data and log ***K_p_***^exp^ values were available. The resulting dependences between the calculated and experimental log ***K_p_*** values were linear, with R^2^ = 0.97, 0.94 and 0.98, respectively. However, when eight additional, nonsteroid compounds (mainly drugs of low to medium lipophilicity, not particularly bulky molecules, with moderate ability to form H-bonds) **39** to **46** (ibuprofen, salicylic acid, indomethacin, naproxen, methylparaben, aspirin, piroxicam, and ranitidine) were incorporated in a test set, the correlations between the calculated and experimental log ***K_p_*** values remained linear only for Equation (4), with R^2^ = 0.85 (for Equation (3) and Equation (5) R^2^ = 0.53 and 0.30, respectively). 

The result obtained for Equation (4) (as compared to Equations (3) and (5)) confirms the versatility of Equation (4) which was tested on a set of compounds of different physicochemical properties. It is stressed here that the coefficients for ***PSA***, ***HD*** and ***V_M_*** in Equation (4) are negative (as opposite to Equations (3) and (5)) which (as already observed, e.g., by Lien and Gaot [48]) suggests that excessive hydrogen bonding, polar surface area and molecular size are obstacles to epidermal permeability.

**Figure 6:**
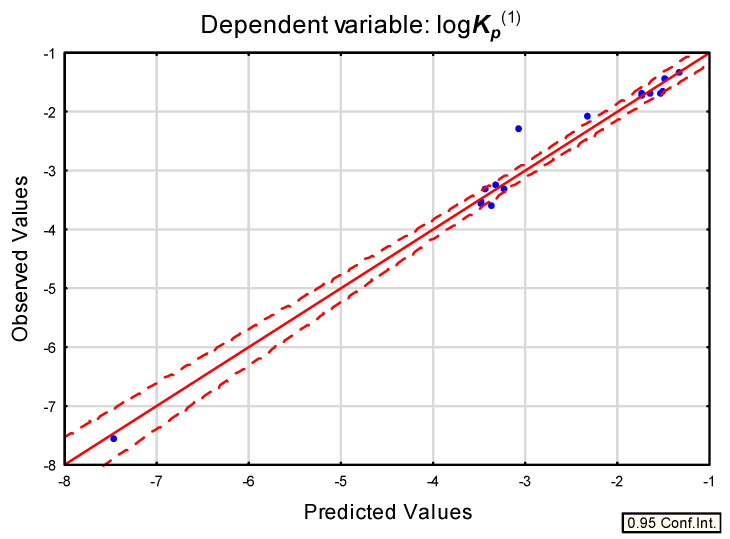
Equation (7) predicted vs. observed values.

Equation (4), efficient as it may be, seems somewhat over-parameterized. In search for a simpler, yet efficient model, Equations (6)–(10) were considered: (Figure 6)

log ***K_p_***^(1)^ = 0.43 (±0.30) − 0.17 (±0.07) ***HD*** − 0.010 (±0.001) ***V_M_*** + 1.48 (±0.17) ***R_M_***

(*n* = 16, R^2^ = 0.98, R^2^_adj_. = 0.97, RMSECV = 0.42, F = 195.2, *p* < 0.01, s_e_ = 0.24) (6)

log ***K_p_***^(1)^ = 0.20 (±0.35) + 1.09 (±0.34) ***R_M_*** − 0.0063 (±0.0025) ***V_M_*** − 0.015 (±0.007) ***PSA***

(*n* = 16, R^2^= 0.98, R^2^_adj_. = 0.97, RMSECV = 0.41, F = 176.0, *p* < 0.01, s_e_ = 0.26) (7)

log ***K_p_***^(1)^ = 0.58 (±0.35) + 1.80 (±0.14) ***R_M_*** − 0.011 (±0.001) ***V_M_***

(*n* = 16, R^2^ = 0.97, R^2^_adj_.= 0.96, RMSECV = 0.31, F = 201.5, *p* < 0.01, s_e_ = 0.29) (8)

log ***K_p_***^(1)^ = −0.14 (±0.16) − 0.035 (±0.002) ***PSA.***

(*n* = 16, R^2^ = 0.96, R^2^_adj_. = 0.96, RMSECV = 0.40, F = 327.3, *p* < 0.01, s_e_ = 0.32) (9)

log ***K_p_***^(1)^ = 0.60 (±0.78) − 0.61 (±0.12) ***HD*** − 0.0079 (±0.0026) ***V_M_***

(*n* = 16, R^2^ = 0.85, R^2^_adj_. = 0.83, RMSECV = 0.69, F = 37.5, *p* < 0.01, s_e_ = 0.64) (10)

Equations (6)–(10) were tested on a set of 13 compounds whose log ***K_p_***^exp^ values were available (compounds **1** to **5** and **39** to **46**), giving correlations of different quality (R^2^ = 0.75, 0.83, 0.67, 0.79 and 0.74, respectively). Equation (7), which is a simplified version of Equation (4) (with one independent variable (***HD***) omitted), gave the best fit with experimental log ***K_p_*** data. However, Equations (9) and (10), unlike other equations developed in this study, do not require access to compound samples, so they have the benefit of applicability, e.g., to new drugs at the design stage. Equation (9), which contains only one independent variable (***PSA***), is somewhat similar to the blood and brain barrier (BBB) permeability and human intestinal absorption (HIA) models developed by Clark [49,50], which strengthens the notion that physicochemical properties associated with good penetration of different biological barriers are interrelated. 

Equations (9) and (10) were tested on a group of all compounds (steroids and nonsteroids) whose log ***K_p_***^exp^ values were available, including solutes that had not been used for validation of other equations because of the lack of chromatographic data. It was established that log ***K_p_*** values calculated according to these equations (log ***K_p_***^(9)^ and log ***K_p_***^(10)^) were in moderate agreement with experimental data for a dataset containing 24 compounds (**1** to **16** and **39** to **46**) (R^2^ = 0.65 and 0.62), but correlations were poorer for the group of hydrocortisone esters **17** to **27** studied by Anderson [40]. It was, therefore, concluded that Equations (9) and (10) should be used with caution for rapid, rough estimations of skin permeability of compounds before they are synthesized. In other situations, predictions based on more sophisticated models (e.g., Equations (1) or (7)) are recommended. 

## 3. Materials and Methods

### 3.1. Chemicals

The 16 steroid drugs analyzed experimentally during these investigations (**1** to **16**: cortisol, hydrocortisone acetate, methyltestosterone, progesterone, testosterone propionate, testosterone heptanoate, cortisone acetate, prednisolone, estrone, estradiol benzoate, desoxycorticosterone acetate, tibolone, spironolactone, eplerenone, digoxin and dexamethasone) were donated as free samples by Polfa-Pabianice or isolated from pharmaceutical preparations. Nonsteroid compounds **39** to **46** (ibuprofen, salicylic acid, indomethacin, naproxen, methylparaben, aspirin, piroxicam, and ranitidine) were also donated as free samples by Polfa-Pabianice or isolated from pharmaceutical preparations. The purity of solutes isolated from pharmaceutical preparations was assessed by thin layer chromatography and densitometry. All isolated compounds gave single chromatographic spots (densitometric peaks) and were used without further purification. Compounds obtained from Polfa-Pabianice were of analytical or pharmacopeial grade. Distilled water used for chromatography was obtained from an in-house distillation apparatus. Analytical grade acetonitrile and methanol were obtained from Avantor Performance Materials (formerly Polskie Odczynniki Chemiczne, Gliwice, Poland). pH 7.4 phosphate buffered saline was obtained from Sigma-Aldrich. 

### 3.2. Thin Layer Chromatography

Thin layer chromatography was performed according to [33] on 10 × 20 cm glass-backed RP-18 F_254s_ TLC plates from Merck, Germany (layer thickness 0.25 mm). Before use, the plates were prewashed with methanol-dichloromethane 1:1 (*v*/*v*) and dried overnight in ambient conditions. Solutions of compounds **1** to **16** in methanol (1 μg·μL^−1^, spotting volume 1 μL), were spotted with a Hamilton microsyringe 15 mm from the plate bottom edge, starting 10 mm from the plate edge, at 8 mm intervals. The chromatographic plates were developed in a vertical chromatographic chamber lined with filter paper and previously saturated with the mobile phase vapor for 20 min. The mobile phase consisted of acetonitrile/pH 7.4 phosphate buffered saline 70:30 (*v*/*v*). The development distance was 95 mm from the plate bottom edge. After development, the plates were dried at room temperature and examined under UV light (254 nm) and with the Desaga CD60 densitometer (Multiwavelength Scan, 200–300 nm at 20 nm intervals). All chromatograms were repeated in duplicate, and the mean ***R_f_*** values were used in further investigations. The chromatographic parameter ***R_M_*** considered in these investigations was defined by Bate-Smith and Westall: ***R_M_***= log (1/***R_f−_***_1_) [51]. The chromatographic data are presented in Table 2.

### 3.3. Calculated Molecular Descriptors

The molecular descriptors for compounds investigated during this study (octanol water partition coefficient log ***P_ow_***; molecular weight ***M_W_***; distribution coefficient log***D***; polar surface area ***PSA***; H-bond donors count ***HD***; H-bond acceptors count ***HA***; freely rotatable bonds count ***FRB***; molar volume ***V_M_***; polarizability ***α***; molar refractivity ***R***) were calculated using ACD/Labs 8.0 software. Total oxygen and nitrogen atom count (***N + O***) was calculated from molecular formulae. The calculated molecular descriptors are given in Table 2. Statistical analysis was done using Statistica v.13 or StatistiXL v. 2. Equations (3)–(10) were tested using leave-one-out methodology. 

## 4. Conclusions

The skin permeability of steroids, as investigated in this study, is important because some of these compounds are, or could be used in preparations applied topically. Predicting skin permeability of steroids is a difficult task because steroid drugs have very different physicochemical properties and may cross the skin barrier by a variety of mechanisms [4]. Experimental skin permeability data exist only for a part of the studied group and they form three mutually incompatible steroid datasets [1,4], with experimental values given by Anderson et al. [40] distinctively higher than expected, as already reported by Abraham et al. [4]. Due to the limited availability of consistent experimental data for the studied solutes, the reference skin permeability coefficients log ***K_p_*** were calculated using three methods: log ***K_p_***^EPI^ based on log ***P_ow_*** and ***M_w_*** as proposed by Potts and Guy [10]; Equation (1) developed earlier [36] and based on (***N + O***), ***HD*** and log ***D***; and by preADMET software [38]. It was established that Equation (1), proposed for structurally unrelated, nonsteroid drugs was also applicable to the group of studied steroids, as shown using a subset of compounds whose experimental log ***K_p_*** data were available. It is also a useful tool to study the partition between the stratum corneum (especially the lipid domain) and water. However, the solutes from the so-called “Anderson dataset” [40] form a separate subgroup, parallel to the correlation line obtained for compounds studied by other authors [1,2] (Figure 1 and Figure 2). Skin permeability models developed earlier (Equation (1) [36]) or in this study (Equations (4), (7), (9) and (10)) were found to predict log ***K_p_*** of steroids fairly well (especially Equations (1) and (7)) and have the benefit of being based only on calculated descriptors (Equations (1), (9) and (10)). It was established that the applicability of equations proposed in this study ((7), (9) and (10)) extend beyond steroid compounds.

## Figures and Tables

**Table 1:** Calculated and experimental log ***K_p_*** values for compounds **1** to **46**.

	log*K_p_*^EPI^	log*K_p_*^pre^	log*K_p_*^exp^	log*K_p_*^(1)^	log*K_p_*^(3)^	log*K_p_*^(4)^	log*K_p_*^(5)^	log*K_p_*^(6)^	log*K_p_*^(7)^	log*K_p_*^(8)^	log*K_p_*^(9)^	log*K_p_*^(10)^
**1**	−3.72	−3.88	−4.19	−3.55	−3.75	−3.57	−4.06	−3.57	−3.47	−3.30	−3.46	−3.56
**2**	−3.77	−4.71	−4.79	−3.62	−3.65	−3.45	−4.22	−3.39	−3.35	−3.10	−3.46	−3.44
**3**	−2.00	−2.15	−1.36	−1.47	−1.87	−1.48	−2.04	−1.57	−1.48	−1.51	−1.33	−1.68
**4**	−3.75	−4.24	−4.35	−3.25	−3.63	−3.41	−4.29	−3.33	−3.31	−3.03	−3.46	−3.39
**5**	−2.24	−2.49	−2.44	−1.75	−2.49	−1.80	−2.64	−1.94	−1.73	−1.82	−1.44	−1.84
**6**	−4.11	−4.42	−4.41	−3.54							−3.07	−2.76
**7**	−3.46	−4.23	−3.26	−2.98							−2.75	−2.86
**8**	−1.78	−2.35	−2.82	−1.62							−1.44	−2.30
**9**	−2.55	−2.97	−3.22	−2.23							−2.04	−2.26
**10**	−2.20	−3.42	−3.22	−2.38							−2.15	−2.88
**11**	−2.74	−3.42	−3.34	−2.15							−2.04	−2.27
**12**	−2.22	−2.54	−2.65	−1.78							−1.44	−2.03
**13**	−2.70	−3.90	−4.12	−3.29							−2.75	−4.07
**14**	−1.67	−2.72	−2.21	−1.91							−1.56	−2.45
**15**	−2.80	−4.05	−4.39	−2.68							−2.26	−3.04
**16**	−3.85	−4.54	−5.00	−3.38							−3.35	−2.83
**17**	−4.44	−4.42	−4.59	−4.90							−5.18	−4.61
**18**	−4.20	−4.24	−4.17	−4.35							−4.38	−3.67
**19**	−3.75	−3.53	−3.68	−4.27							−4.59	−3.54
**20**	−4.00	−3.63	−3.20	−4.59							−4.98	−3.95
**21**	−3.47	−2.45	−2.74	−4.43							−4.98	−4.33
**22**	−4.10	−3.43	−3.05	−4.26							−4.38	−4.05
**23**	−3.63	−3.11	−3.04	−4.13							−4.38	−4.23
**24**	−3.29	−3.26	−2.47	−3.49							−3.67	−3.26
**25**	−3.26	−2.35	−2.27	−4.12							−4.59	−3.92
**26**	−2.41	−1.82	−1.74	−3.24							−3.67	−3.64
**27**	−1.90	−1.35	−1.21	−3.08							−3.67	−3.90
**28**	−1.28	−2.33		−1.71	−1.25	−1.63	−2.07	−1.58	−1.51	−1.28	−1.77	−2.51
**29**	−3.62	−4.13		−3.57	−3.69	−3.42	−4.24	−3.30	−3.46	−3.15	−3.67	−3.14
**30**	−2.85	−2.81		−2.10	−2.67	−2.19	−2.79	−2.21	−2.30	−2.25	−2.25	−1.96
**31**	−3.67	−3.35		−3.34	−3.89	−3.24	−4.30	−3.11	−3.40	−3.14	−3.56	−2.52
**32**	−1.58	−2.03		−1.69	−1.56	−1.48	−2.19	−1.44	−1.49	−1.32	−1.66	−1.85
**33**	−2.27	−2.19		−1.70	−1.99	−1.74	−2.18	−1.87	−1.63	−1.68	−1.44	−2.16
**34**	−0.58	−1.28		−1.36	−0.72	−1.36	−1.46	−1.30	−1.31	−1.07	−1.66	−2.36
**35**	−3.64	−3.68		−2.31	−3.53	−2.81	−3.76	−2.73	−3.05	−2.86	−3.14	−2.05
**36**	−4.10	−4.19		−3.34	−4.09	−2.96	−3.70	−3.04	−3.20	−3.27	−2.90	−1.89
**37**	−6.35	−4.98		−7.56	−6.34	−7.54	−5.15	−7.68	−7.44	−7.35	−7.25	−7.57
**38**	−1.95	−1.32		−1.70	−2.16	−1.83	−2.17	−2.01	−1.73	−1.85	−1.44	−2.17
**39**			−1.44		−2.39	−1.60	−2.99	−1.64	−1.54	−1.48	−1.44	−1.59
**40**			−4.05		−4.69	−3.73	−5.31	−3.55	−3.86	−3.53	−4.05	−2.71
**41**			−2.14		−3.63	−2.08	−4.80	−1.88	−2.18	−1.86	−2.37	−1.11
**42**			−2.04		−3.20	−1.71	−4.23	−1.60	−1.73	−1.53	−1.77	−0.99
**43**			−2.84		−2.91	−1.77	−4.79	−1.47	−1.70	−1.19	−2.15	−1.41
**44**			−3.67		−3.29	−2.56	−3.62	−2.55	−2.62	−2.51	−2.58	−2.13
**45**			−3.81		−3.13	−2.68	−5.72	−2.04	−2.76	−1.75	−3.92	−2.29
**46**			−2.54		−2.94	−1.89	−3.48	−1.90	−1.88	−1.82	−1.77	−1.52

**Table 2:** Physicochemical and chromatographic descriptors for compounds **1** to **46**.

		log *P*	*M_W_*	*PSA*	*FRB*	*HD*	*HA*	*R*	*V_M_*	*α*	*N + O*	log*D*	*R_M_*
**1**	Dexamethasone	1.87	392.5	94.8	5	3	5	100.2	296.2	39.7	5	1.87	−0.35
**2**	Hydrocortisone (HC)	1.43	362.5	94.8	5	3	4	95.6	281.4	37.9	5	1.43	−0.33
**3**	Progesterone	4.04	314.5	34.1	1	0	2	91.0	289.0	36.6	2	4.04	0.60
**4**	Prednisolone	1.49	360.4	94.8	5	3	5	95.5	274.7	37.9	4	1.49	−0.33
**5**	Estrone	3.69	270.4	37.3	1	1	2	78.1	232.2	30.9	2	3.69	0.09
**6**	Aldosterone	0.46	360.4	83.8	4	2	5	93.7	272.1	37.1	5	0.46	
**7**	Corticosterone	1.76	346.5	74.6	4	2	4	94.0	284.3	37.3	4	1.76	
**8**	Pregnenolone	4.52	316.5	37.3	2	1	2	92.4	290.0	36.6	2	4.52	
**9**	17-α-Hydroxyprogesterone	2.89	330.5	54.4	2	1	3	92.6	286.1	36.7	3	2.89	
**10**	17-α-Hydroxypregnenolone	3.38	332.5	57.5	3	2	3	93.9	287.2	37.2	3	3.38	
**11**	Deoxycorticosterone	3.41	330.5	54.4	3	1	3	92.5	286.3	36.7	3	3.41	
**12**	Testosterone	3.48	288.4	37.3	1	1	2	83.1	257.0	33.0	2	3.48	
**13**	Cortexolone	1.74	346.5	74.6	2	4	2	94.1	283.4	37.3	4	2.74	
**14**	Estradiol	4.13	272.4	40.5	2	2	2	79.5	232.6	31.5	2	4.13	
**15**	Estriol	2.94	288.4	60.7	3	3	3	81.1	229.6	32.2	3	2.94	
**16**	Cortisone	1.44	360.4	91.7	4	2	5	94.2	280.3	37.3	5	1.44	
**17**	HC succinamate	1.45	461.6	144.0	9	4	8	118.2	351.8	46.8	8	1.45	
**18**	HC N,N-dimethylsuccinate	2.05	489.6	121.2	9	2	8	127.7	386.8	50.6	8	2.05	
**19**	HC methylsuccinate	2.53	476.6	127.2	10	2	8	120.9	370.4	47.9	8	2.53	
**20**	HC hemisuccinate	2.13	462.5	138.2	9	3	8	116.1	345.6	46.0	8	1.95	
**21**	HC pimelate	3.07	504.6	138.2	12	3	8	130.0	393.9	51.5	8	2.99	
**22**	HC pimelamate	2.61	531.7	121.2	12	2	8	141.6	435.0	56.1	8	2.61	
**23**	HC 6-hydroxyhexanoate	2.63	476.6	121.1	12	3	7	125.2	381.0	49.6	7	2.63	
**24**	HC propionate	3.05	418.5	100.9	7	2	6	109.8	335.4	43.5	6	3.04	
**25**	HC methylpimelate	3.53	518.6	127.2	13	2	8	134.8	418.7	53.4	8	3.53	
**26**	HC hexanoate	4.64	460.6	100.9	10	2	6	123.7	383.7	49.0	6	4.64	
**27**	HC octanoate	5.70	488.7	100.9	12	2	6	132.9	415.9	52.7	6	5.70	
**28**	Estradiol benzoate	6.24	376.5	46.53	4	1	3	109.3	317.6	43.3	3	6.24	0.91
**29**	HC acetate	2.51	404.5	100.9	6	2	6	105.2	319.3	41.7	6	2.51	−0.12
**30**	Deoxycortisone acetate	4.53	372.5	60.4	4	0	4	102.1	324.3	40.5	4	4.53	0.41
**31**	Cortisone acetate	2.53	402.5	97.7	5	1	6	103.8	318.2	41.1	6	2.53	−0.12
**32**	Testosterone propionate	4.90	344.5	43.4	3	0	3	97.3	311.2	38.6	3	4.90	0.85
**33**	Methyltestosterone	4.02	302.5	37.3	1	1	2	87.8	273.0	34.8	2	4.02	0.41
**34**	Testosterone enanthate	7.03	400.6	43.4	7	0	3	115.9	375.9	45.9	3	7.03	1.38
**35**	Spironolactone	3.12	416.6	85.7	2	0	4	112.7	335.8	44.7	4	3.12	0.14
**36**	Eplerenone	1.05	414.5	78.9	2	0	6	106.1	315.7	42.1	6	1.05	−0.21
**37**	Digoxin	0.85	780.9	203.1	13	6	14	196.4	572.3	77.9	14	0.85	−0.91
**38**	Tibolone	4.02	312.5	37.3	1	1	2	90.0	274.2	35.7	2	4.02	0.33
**39**	Ibuprofen			37.3		1			200.3				0.08
**40**	Ranitidine			111.6		2			265.5				−0.66
**41**	Aspirin			63.6		1			139.6				−0.50
**42**	Methylparaben			46.5		1			124.8				−0.41
**43**	Salicylic acid			57.5		2			100.4				−0.37
**44**	Indomethacin			69.6		1			269.6				−0.07
**45**	Piroxicam			108		2			212.0				0.00
**46**	Naproxen			46.5		1			192.3				−0.16

## Data Availability

The data presented in this study are available in this manuscript.
